# Prevalence of Neuropathic Pain in Morocco: A Systematic Review and Meta-Analysis

**DOI:** 10.3390/life15050780

**Published:** 2025-05-14

**Authors:** Zhor Zeghari, Jihane Belayachi, Redouan El Ouardi, Redouane Abouqal

**Affiliations:** 1 Laboratory of Biostatistics, Clinical and Epidemiological Research, Faculty of Medicine and Pharmacy—Mohammed V University, Rabat 10000, Moroccor.abouqal@um5s.net.ma (R.A.); 2 Acute Medical Unit, Ibn Sina University Hospital, Rabat 10000, Morocco; 3 Bottu Pharmaceuticals, Casablanca 20580, Morocco

**Keywords:** neuropathic pain, prevalence, meta-analysis, Morocco

## Abstract

This study aims to assess the prevalence of neuropathic pain in the Moroccan population through a systematic review and meta-analysis using a generalized linear mixed model (GLMM). The PRISMA guidelines for systematic reviews and meta-analysis were followed. All observational prevalence studies, conducted in both the general population and hospital settings in Morocco, published before 1 December 2023, were included, provided that neuropathic pain was identified based on consensus criteria. The databases searched included PubMed, Scopus, Web of Science, and the gray literature (Google Scholar). Data on the sample size, subgroups, sociodemographic and clinical characteristics of participants, as well as the methodology of each study, were extracted. The Joanna Briggs Institute tool for prevalence studies was used to assess the risk of bias. A random-effects generalized linear mixed model was applied for direct data adjustment, using Knapp–Hartung standard error correction. Heterogeneity was explored using Cochran’s Q test, Higgins’ I^2^ statistic, and prediction intervals. Subgroup analysis was performed based on underlying pathology, while meta-regression was conducted according to age and sex ratio. Publication bias was assessed using the Doi plot and LFK test. Sensitivity analysis was performed by stratifying studies based on their risk of bias. The statistical analysis was conducted using R software version 4.3.1 with the meta, metafor, metasens, and robvis packages. A total of 33 publications were identified, of which 17 studies were retained after removing duplicates and applying the eligibility criteria. These studies were published between 2013 and 2023 and included either the general population (one study) or patients with diabetes (seven), obesity (one), rheumatologic conditions (six), Parkinson’s disease (one), or cancer (one). The DN4 score was the most commonly used tool to diagnose neuropathic pain. The risk of bias was rated as low in two studies, moderate in eight, and high in seven. The pooled overall prevalence was 22% (95% CI [14.8; 31.5]) with an I^2^ of 98% (*p* < 0.01). In subgroup analyses, the prevalence was 19.9% among rheumatology patients, 10.2% in oncology patients, 9.4% in Parkinson’s patients, 26.6% in diabetics, 58.6% in obese individuals, and 10.6% in the general population. Meta-regression by sex indicated significantly lower prevalence in men, and prevalence increased with age but did not reach statistical significance. After stratification based on the risk of bias, the pooled prevalence was 15.4% for the two studies with a low risk of bias. The overall prevalence of neuropathic pain in Morocco is relatively high at 22%, with significant variability across medical specialties. However, this prevalence is likely overestimated. Stronger and higher-quality studies are needed to obtain more accurate estimates.

## 1. Introduction

Neuropathic pain is defined by the IASP (International Association of the Study of Pain) as pain resulting directly from a lesion or disease affecting the somato-sensory system [1,2].

A variety of pathological mechanisms are in cause, and it is generally classified according to its anatomical location or underlying cause. The main factors and pathophysiological conditions associated with the development of neuropathic pain include metabolic disorders such as diabetic peripheral neuropathy, neuropathies linked to viral infections such as post-herpetic neuralgia, autoimmune diseases affecting the central nervous system such as multiple sclerosis, peripheral neuropathies caused by chemotherapy, trauma-induced nerve damage (spinal cord injury and amputation), inflammatory conditions and hereditary neuropathies [2,3,4]. This type of pain occurs spontaneously without any stimulus, and is usually associated with allodynia, hyperalgesia and paresthesia. It tends to be chronic and consequently deeply affects the quality of life of patients compromising their psychological state [1,4,5].

This condition is widely recognized as one of the most difficult pains syndromes to manage and outcomes often are unsatisfactory. The disparity between the clinical picture and response to analgesics calls for specific analgesic management. Tricyclic antidepressants, gabapentin, pregabalin, and serotonin noradrenaline reuptake inhibitors are recommended as first-line drugs [2,3,4].

However, neuropathic pain is often under-diagnosed, leading to under-treatment or poor management, partly because the neuropathic aspect of pain is not always recognized in primary care consultations. In the past decade, the creation of simple, targeted questionnaires focusing on patients’ verbal descriptions of the qualitative aspects of pain has enhanced both its diagnosis and treatment [6]. As validated self-administered questionnaires to identify neuropathic pain have been developed, these have enabled epidemiological studies. More and more studies explore the epidemiology of neuropathic pain. Estimates of neuropathic pain prevalence are essential in order to determine resource requirements for health care, and to inform the targeting of treatment and prevention strategies [7]. The neuropathic pain prevalence varies considerably in the literature, with estimates ranging from 0.9% to 17.9% depending on the methods and populations used [8]. Prevalence figures must be adapted to the populations concerned. *The limited number of reliable data concerning the prevalence of neuropathic pain in Morocco and in the region is the main motivation that prompted us to assess this prevalence in the Moroccan population through a systematic review and meta-analysis. Then, this study aimed to assess the prevalence of neuropathic pain in the Moroccan population through a systematic review and meta-analysis*.

## 2. Method

The PRISMA 2020 guidelines were followed in the conduct and reporting of this systematic review and meta-analysis [9]. A protocol of this study was previously registered in the OSF database (https://doi.org/10.17605/OSF.IO/EV4HA (accessed on 7 April 2025).

### 2.1. Data Sources

We undertook a comprehensive electronic search across major databases including PubMed Web of Science and Scopus to identify relevant studies. The search terms comprised combinations of MESH terms and free words. Additionally, we explored the gray literature via Google Scholar, medical thesis registers and conference papers. Furthermore, we traced the citations of identified articles and scanned the reference lists of review papers and conference proceedings. The last search data were obtained on 30 December 2023.

### 2.2. Screening Strategy and Inclusion Criteria

Two investigators (ZZ and JB) independently screened all citations by title and abstract, and then by full text. The irrelevant studies and duplicates were removed manually, and where multiple papers were generated from the same data, only the most relevant paper was included. Disagreements were resolved by consensus or by consulting a third investigator (RA). The studies included had to fulfil the following criteria.

(1) Population- or hospital-based cross-sectional studies; (2) studies involving adults in Morocco; (3) studies reporting the prevalence of NP overall and by different subgroups of interest, according to any of the internationally accepted diagnostic criteria for NP, or those providing enough data to estimate this prevalence; and (4) studies published in English or French. No restrictions were placed on the sample size or sampling methods.

### 2.3. Search Strategy

The key words were “neuropathic pain”, “prevalence” and “Morocco”. These terms’ synonyms were searched, translated into medical subject headings (MeSH) and combined with “OR” and “AND” operators. Free-text terms were used for the gray literature. The search string was first developed for PubMed and then later adapted for the other databases (Supplementary File S1). The search strategy was tested across the selected databases and approved by the senior reviewer (RA) before the screening. A database record was maintained at each step of the review process detailing how the search was undertaken, including the results of the search strategy.

### 2.4. Data Extraction

Relevant data for this review were extracted using a purposeful design and a piloted extraction form. The information extracted included (1) author details [names and year of publication]; (2) study characteristics [city, study design, setting, data source, sampling method, sample size, and data collection period]; (3) participants’ characteristics [age, gender, sample size, city, and NP-related factors [context of diagnosis, specialty, and causative condition]; and (4) NP characteristics [diagnostic criteria, diagnostic threshold, and number of participants tested and diagnosed with NP overall and by subgroups of interest].

### 2.5. Quality Assessment

The quality of included studies was independently appraised by two reviewers (ZZ and JB). Disagreements were resolved through discussion with the senior reviewer (RA). The Joanna Briggs Institute (JBI) Critical Appraisal tool for systematic reviews of observational epidemiological studies reporting prevalence and incidence data was used. The nine items of the Checklist for Prevalence Studies were scored as follows: a response of “Yes” was assigned a score of 1, while responses of “No” or “Unclear” were assigned a score of 0. The overall risk of bias was classified as high, moderate, or low based on the total score: high risk of bias: 0–3 criteria met (low-quality study); moderate risk of bias: 4–6 criteria met (moderate-quality study); low risk of bias: 7–9 criteria met (high-quality study). This classification allowed for a standardized assessment of study quality and potential biases in the included prevalence studies [10].

### 2.6. Statistical Analysis

The aim of a meta-analysis of proportion is to estimate the overall proportion regardless of the transformation used. Generalized linear mixed models (GLMMs) are ideal for combining proportions because they do not require corrections for zero counts and they fully account for within-study uncertainties, especially for small sample sizes and rare events. This is why a GLMM model was used to directly fit the data using the exact binomial likelihood, with a Hartung Knapp adjustment.

The pooled prevalence with a 95% CI was reported as a summary measurement, with the results being presented as a forest plot. Heterogeneity was explored with Cochran’s statistics, Higgins’ I2 and the prediction interval. Subgroup analysis was performed according to underlying pathology, and meta-regression according to age and sex ratio. Publication bias were explained using the Doi plot and LFK test. Sensitivity analysis was performed by stratifying on risk of study bias.

Data were analyzed using R software 4.3.1 with the packages meta, metafor, metasens and robvis.

### 2.7. Missing Data

Additional information was collected by contacting the authors of the included studies with missing data.

## 3. Results

**Figure 1:**
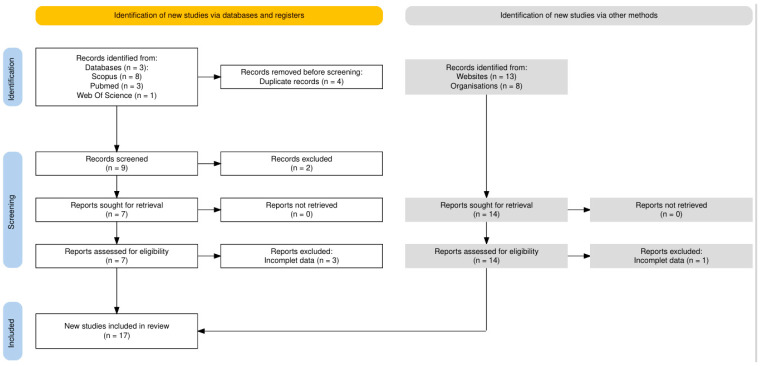
Flow chart of the search strategy.

The search yielded 33 records, of which 22 were retained after removing duplicates. After reviewing the abstracts and titles, two studies were found not to meet the inclusion criteria. Out of the 20 studies that underwent a full-text review, 17 papers were deemed eligible. A PRISMA diagram illustrating the search strategy is shown in Figure 1.

### 3.1. Characteristics of Included Studies

Six university hospital centers were represented by Marrakech [11,12,13,14,15,16,17,18], Rabat [19,20], Fes [21,22], Tanger [23], Oujda [24].and Casablanca [25] The majority of studies involved patients followed in rheumatology [14,15,18,21,23,24] or diabetology departments [12,16,17,22,25,26,27], but some also involved patients in oncology [19], patients in neurology [20] or those followed for obesity [13]. One study was population wise [11]. A total of 11,413 individuals were analyzed, with study sizes ranging from 21 to 5116 participants. The mean age was between 38 and 63 years. Roughly 70% of respondents were women. The DN4 score was used to diagnose neuropathic pain in 15 of the 17 studies, but only 3 used a cross-culturally adapted version [11,15,23]. The score cut off was 4 for 13 studies and 3 for the rest. The characteristics of the 17 included studies are summarized in Table 1.

### 3.2. Quality Assessment

**Figure 2:**
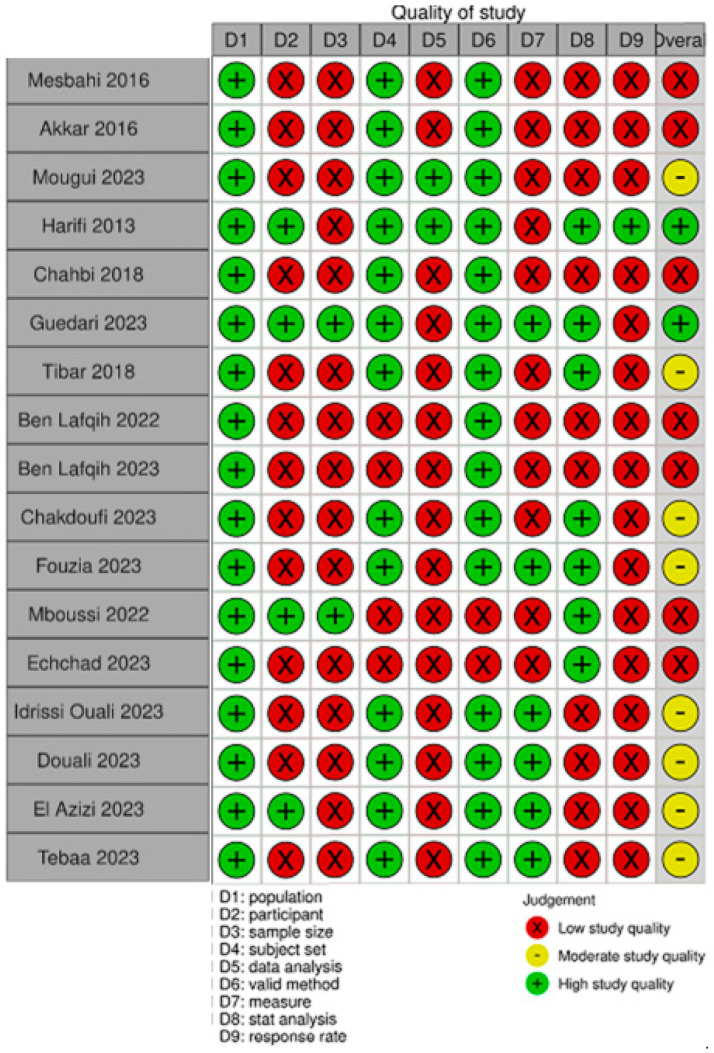
The JBI critical appraisal tool for cross-sectional prevalence studies by study. Figure presents the risk of bias assessment for each study included in the systematic review and meta-analysis. The bias assessment criteria (D1 to D9) are listed in the columns and include the following: D1: population; D2: participant; D3: sample size; D4: subject selection; D5: data analysis; D6: valid method; D7: measurement; D8: statistical analysis; D9: response rate. Each criterion is judged based on three levels of risk: green (+): high-quality study (low risk of bias); red (×): low-study quality (high risk of bias); yellow (-): moderate study quality (moderate risk of bias). The overall risk of bias assessment for each study is provided in the last column. This analysis helps to identify the methodological strengths and limitations of the included studies [11,12,13,14,15,17,18,19,20,21,22,23,24,25,26,27].

**Figure 3:**
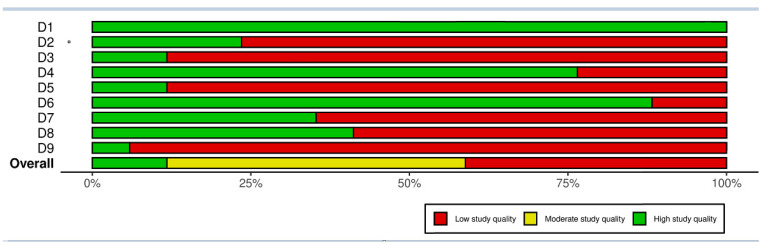
The JBI critical appraisal tool for cross-sectional prevalence studies by item. Assessment of methodological quality using the Joanna Briggs Institute (JBI) critical appraisal tool for cross-sectional prevalence studies. Each row represents a specific appraisal criterion, including population, participant selection, sample size, subject set, data analysis, validity of methods, measurement, statistical analysis, and response rate. The overall assessment is shown at the bottom. The bar chart displays the proportion of studies rated as; green: high-quality studies (low risk of bias); yellow: moderate-quality study (moderate risk of bias); red: low-quality study (high risk of bias).

Figure 2 and Figure 3 show the quality assessment of the included studies using the JBI critical appraisal tool for cross-sectional prevalence studies. Two studies had a high score of good quality [11,25], eight had a moderate one [15,17,18,20,21,22,23,26] and seven had a low one [12,13,14,16,19,24,27].

### 3.3. Overall Prevalence

**Figure 4:**
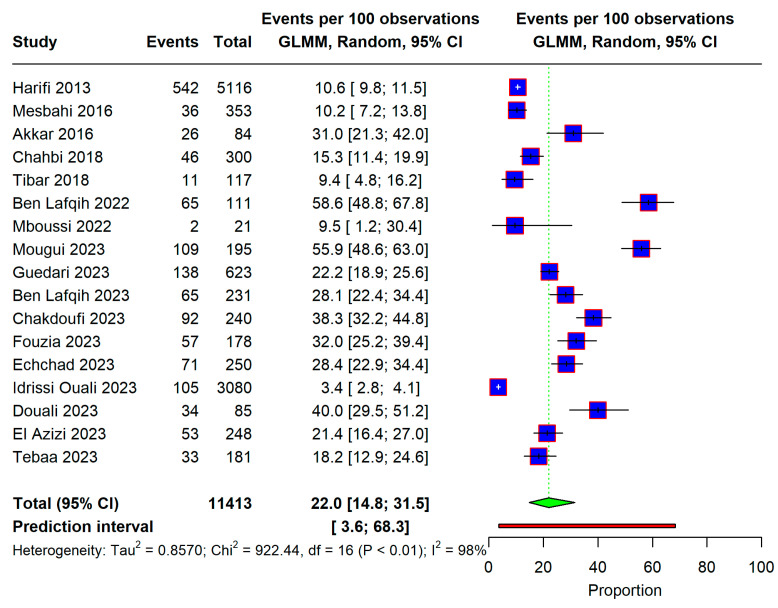
Forest plot showing the overall prevalence of neuropathic pain in Morocco. Forest plot displaying the proportion of events per 100 observations across multiple studies. Each study is represented by a blue square, with the size of the square reflecting the weight of the study. Horizontal lines indicate the 95% confidence intervals (CIs). The green diamond at the bottom represents the overall estimated proportion with its 95% CI. Heterogeneity statistics are provided at the bottom, including Tau^2^, Chi^2^, degrees of freedom (df), and I^2^, which indicate variability across studies. The prediction interval (shown in brackets) estimates the expected range for future studies [11,12,13,14,15,16,17,18,19,20,21,22,26,27].

The analysis included 17 studies. The overall prevalence of neuropathic pain in Morocco was found to be 22.0% [95% CI: 14.8–31.5]; however, significant heterogeneity was found (I^2^ 98%), as shown in Figure 4.

### 3.4. Subgroup Analysis and Meta-Regression

**Figure 5:**
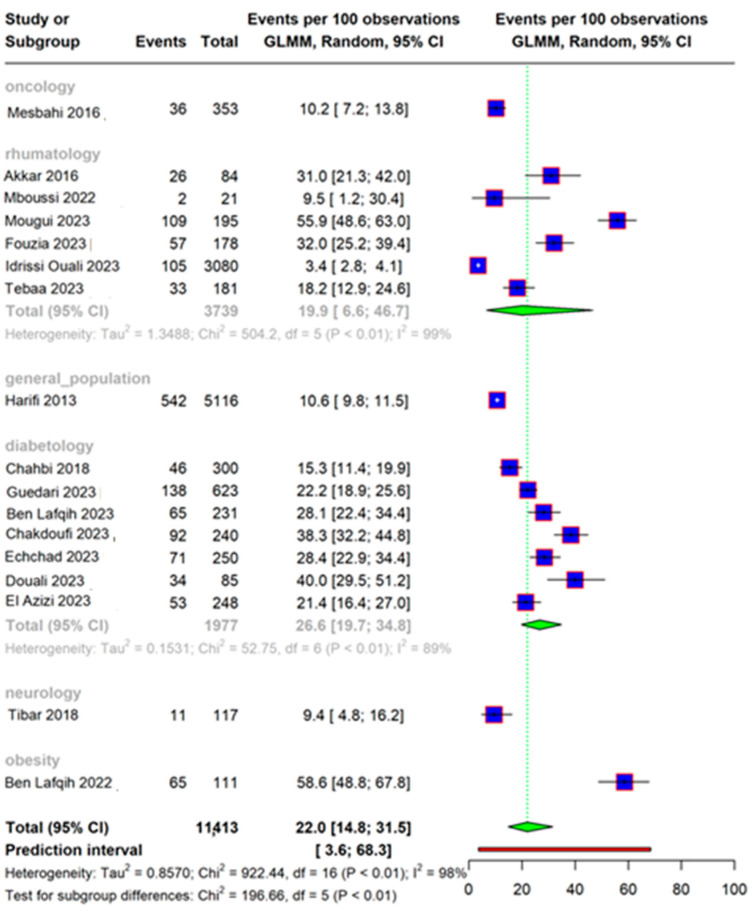
Forest plot showing the subgroup analysis of the prevalence of neuropathic pain. Forest plot displaying the proportion of events per 100 observations (with their 95% confidence intervals) under a random-effects (GLMM) model. The studies are grouped by category (oncology, rheumatology, general population, diabetology, neurology). Each blue square represents the point estimate for a single study, and the square size reflects the study’s weight in the meta-analysis. Horizontal lines indicate the 95% confidence intervals. Green diamonds show the pooled estimates for each subgroup and the overall combined estimate. Heterogeneity statistics (Tau^2^, Chi^2^, I^2^) and the prediction interval (shown at the bottom) illustrate the variability among the studies [11,12,13,14,15,16,17,18,19,20,21,22,26,27].

**Figure 6:**
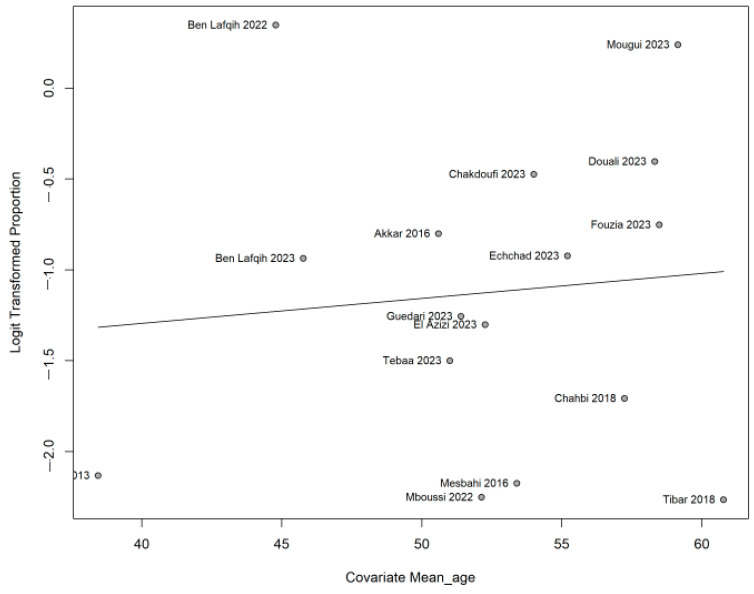
Meta-regression according to age. Meta-regression of logit-transformed proportions by mean age. Each point represents an individual study, labeled with the first author and publication year. The horizontal axis shows the mean age of participants in each study, while the vertical axis shows the logit-transformed proportion of the outcome. The solid line represents the estimated regression line, illustrating the relationship between mean age and the logit-transformed proportion across the included studies.

**Figure 7:**
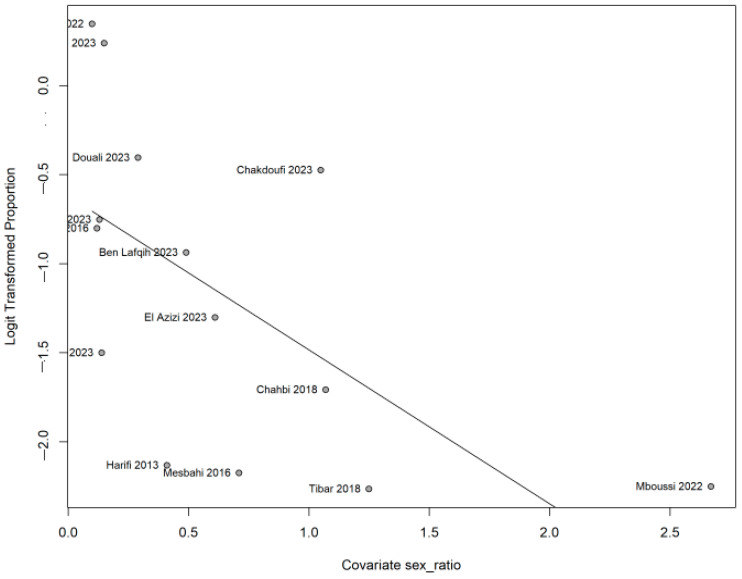
Meta-regression according to sex ratio. Meta-regression assessing the relationship between sex ratio (*x*-axis) and the logit-transformed proportion (*y*-axis). Each point represents a study labeled by the first author and publication year. The solid line indicates the fitted regression, illustrating how the outcome proportion changes with varying sex ratios across studies.

The prevalence of NP seems to vary according to underlying pathology. It reaches 26.6% [95% CI 19.7–34.8] in diabetology patients and 19.9% [95% CI 6.6–46.7] in rheumatology ones, as shown in Figure 5. According to age, the meta-regression did not indicate any change in the prevalence (0.013, *p* 0.693), as shown in Figure 6; however, when the meta-regression was applied to gender, the prevalence was significantly lower in men than women (−0.865, *p* 0.003), as shown in Figure 7.

### 3.5. Publication Bias

**Figure 8:**
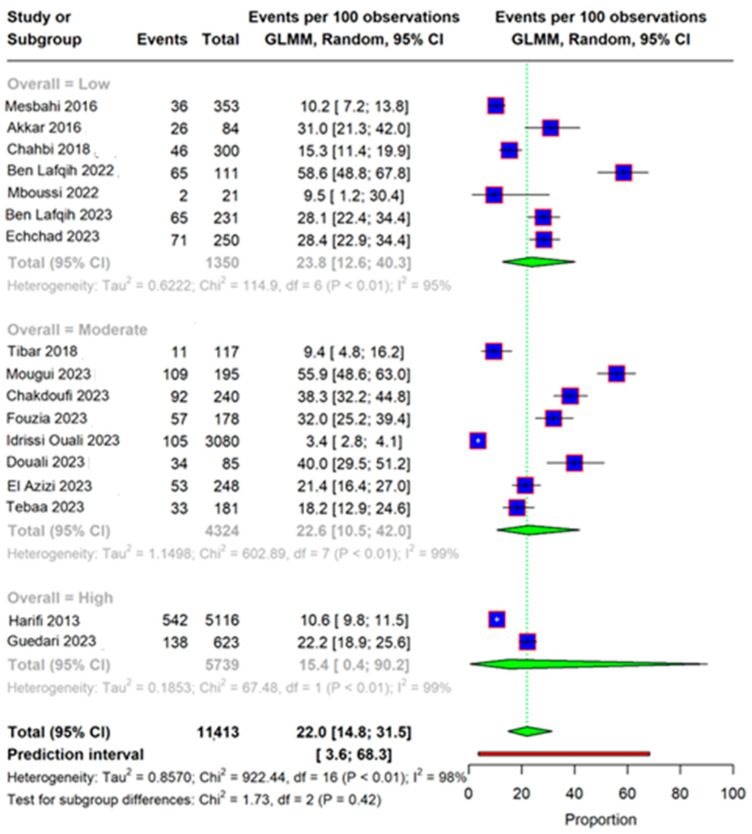
Forest plot showing the pooled prevalence of the outcome across studies, stratified by study quality (Low, Moderate, High). The analysis uses a Generalized Linear Mixed Model (GLMM) with a random-effects approach to estimate the event rate per 100 observations, along with 95% confidence intervals (CI). Each study is represented by a square (proportional to the study weight) and a horizontal line (95% CI). The green diamonds represent the pooled prevalence within each subgroup and overall. Subgroup heterogeneity was evaluated using the I² statistic, showing high variability across and within subgroups. The test for subgroup differences was statistically significant (*p* < 0.01), suggesting that risk of bias may influence prevalence estimates. The prediction interval is shown at the bottom and represents the range within which the true effect of a new study is expected to fall [11,12,13,14,15,16,17,18,19,20,21,22,26,27].

**Figure 9:**
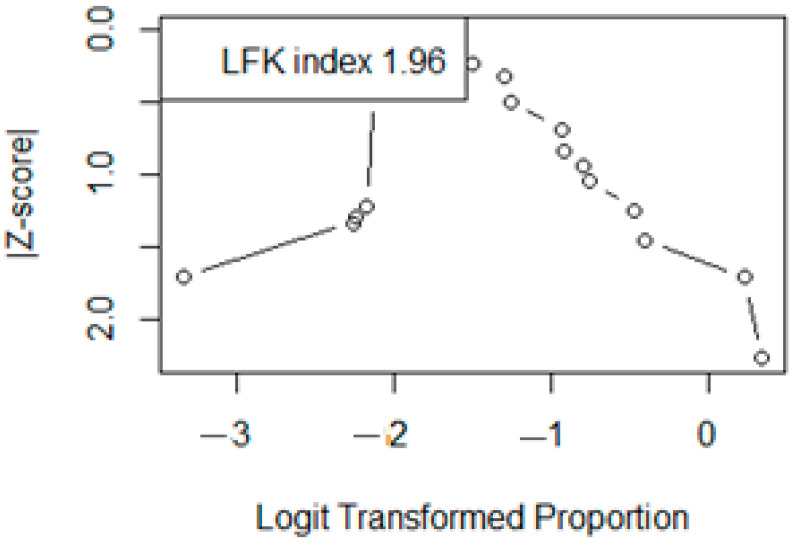
Doi plot assessing publication bias in the meta-analysis using the LFK index. The plot displays the [Z-score] against the logit-transformed proportions for each included study. The LFK index value of 1.96 suggests minor asymmetry, which indicates a slight risk of publication bias. According to guidelines: LFK index within ±1 = no asymmetry, Between ±1 and ±2 = minor asymmetry, Greater than ±2 = major asymmetry. This diagnostic plot complements funnel plots for assessing small-study effects in meta-analyses of proportions.

When subgroups were applied according to quality assessment, sensitivity could not resolve heterogeneity, and the pooled prevalence was significantly lower in the studies of a high quality (15.4% [95% CI 0.4–90.2]), as shown in Figure 8. The DOI plot illustrated in Figure 9 shows an asymmetry with an LFK index of 1.96, which is an indicators of publication bias.

## 4. Discussion

This is the first comprehensive systematic review of epidemiological studies on neuropathic pain prevalence in Morocco. This review was based on observational studies using recently standardized methodology. Overall, the prevalence of neuropathic pain was examined in 17 studies. The pooled prevalence of those reached 22%, was higher among women, and varied significantly with underlying conditions such as diabetes or rheumatological disease, as well as with study quality.

In terms of overall prevalence, our 22% prevalence seems to be higher than that of other studies, although it varies considerably in the literature, with estimates ranging from 0.9% to 17.9% depending on the methods and populations used [7,8]. Several factors can influence the prevalence of neuropathic pain. First of all, the definition of neuropathic pain adopted by each study, and consequently the choice of diagnostic tool for calculating prevalence, may be decisive in determining the value of the pooled prevalence found [6].

In our review, the most common tool to diagnose the neuropathic pain was the DN4 (Douleur Neuropathique en 4 Questions) questionnaire. This self-administered questionnaire is widely used for screening neuropathic pain. It is valued for its simplicity and effectiveness in differentiating neuropathic pain from non-neuropathic pain. The DN4 has been validated and demonstrated high reliability and validity in numerous languages including Turkish, Nepalese and Japanese [28,29,30]. The Moroccan Arabic adapted version of the DN4 was developed in 2011 by Harifi et al. [31]. The DN4 questionnaire is recognized for its high sensitivity and specificity in identifying neuropathic pain. Studies have shown that the DN4 is more sensitive compared to other tools like the LANSS questionnaire, with sensitivities reported to be as high as 95% in some populations [28]. However, its sensitivity can vary depending on the type of neuropathic pain, being the highest for central neuropathic pain and generalized polyneuropathies, and lower for conditions like trigeminal neuralgia [32]. This may explain why, in our review, the prevalence is higher in the diabetic group, diabetic neuropathy being a central pain, than in the rheumatology group, arthropathy being a peripheral pain. The DN4 cut off score used was 4 for most studies, but two studies used a lower cut off score of 3, which probably led to a higher prevalence [11,20].

Moreover, when we examined the studies that make up our review, we found that only the study by Harifi et al. focused on the general population, while the others were hospital-based studies, which are likely to overestimate prevalence. Harifi et al. found a chronic pain with neuropathic characteristics prevalence in Morocco of 10.6% while Van Hecke et al.’s systematic review suggested a prevalence range of 6.9% to 10% for pain with neuropathic characteristics in the general population worldwide [7].

Furthermore, when subgroups were applied, according to the quality assessment in our review, sensitivity could not resolve heterogeneity, and the pooled prevalence was significantly lower in the studies with high quality. This raises the hypothesis that low study quality overestimates prevalence. Our results showed that neuropathic pain was more prevalent in women, which is consistent with the previous evidence. Indeed, the prevalence and intensity of neuropathic pain are higher in females than in males, as this is influenced by a complex interplay of biological, hormonal, and psychosocial factors. These findings underscore the importance of considering gender differences in the diagnosis and treatment of neuropathic pain to improve patient outcomes [33,34]. The two main prevalence subgroups identified in our review were diabetic patients and those with rheumatologic conditions. The prevalence of painful diabetic neuropathy in our review was 26.6%, while it is estimated to be around 20% worldwide [35]. A systematic review and meta-analysis estimated a point prevalence of painful diabetic neuropathy in the Middle East and North Africa (MENA) region of 43.2%, which is notably higher than our estimates and worldwide ones [36].

Across various rheumatic conditions, the prevalence of neuropathic pain is significant, with studies reporting figures around 17% to 21.5% [37]. Our results are consistent with this range, with a prevalence of 19.9%. Neuropathic pain is a common and significant issue in rheumatology, affecting a substantial proportion of patients with conditions like RA, SpA, and PsA. It is associated with higher disease activity, comorbidities, and psychological factors, underscoring the need for comprehensive pain management strategies in these patients. Understanding these associations can aid in better diagnosis and treatment, improving patient outcomes [37,38].

The strengths of this review are that this is the first systematic review and meta-analysis to consider the prevalence of neuropathic pain in Morocco. There are no other systematic studies specifically addressing the prevalence of neuropathic pain in Africa and the Middle East. These initial data show that neuropathic pain is relatively common in Morocco and requires special management.

The main limitation of this systematic search is the limited number of retrieved studies, which can be explained by several factors. Firstly, the use of the “Morocco [Affiliation]” filter restricted results to studies where at least one author was affiliated with a Moroccan institution, potentially excluding relevant studies conducted in Morocco by international research teams without local affiliations. Secondly, the search focused on prevalence studies explicitly mentioning terms such as “nerve pain”, “neuropathy”, or “neuralgia” in the title and abstract fields, which may have led to the omission of studies that used alternative terminologies, such as “chronic pain” or “peripheral neuropathy”. Lastly, while databases like Scopus and Web of Science offer broad indexing criteria, they may have limited coverage of regional journals where Moroccan studies are frequently published; in addition, PubMed primarily indexes the biomedical literature and might not comprehensively capture local epidemiological studies or those published in regional public health and clinical journals. The second issue was the paucity of Moroccan publications on neuropathic pain. Only one study covered the whole population. Four of our studies were abstracts from local conferences, for which we requested the authors’ agreement. The third issue was the low quality of the studies, reviewed which could have led to an overestimation of prevalence.

Following the example of the studies collated in our reviews, our recommendations are that neuropathic pain should be screened in routine practice in diabetes, rheumatology, oncology and neurology consultations, among others. A clear definition with a valid tool in our population should be used, such as the Moroccan Arabic version of the DN4 developed by Harifi et al [31]. More studies on the general population would enable us to better identify the frequency of this pain and its etiologies.

## 5. Conclusions

In summary, our study, the first of its kind in Africa and the Middle East, revealed a prevalence of neuropathic pain in Morocco of 22.0%. This prevalence was higher in women and varied significantly according to underlying conditions and study quality. Further studies in Morocco are needed to broaden the panorama of the frequency of neuropathic pain and its underlying pathologies in the Moroccan population, in order to improve diagnosis and patient management.

## Figures and Tables

**Table 1:** Assessment and prevalence of neuropathic pain of included studies.

Author and Year	City	Specialty	Sample Size	Age Mean ± SD	Score	Cutt off DN4	Cross-Cultural Adaptation	n (%)	Overall Quality
Harifi 2013 [11]	Marrakech	general population	5116	38.45 ± 14.13	DN4	3	Yes	542 (10.6)	high
Mesbahi 2016 [19]	Rabat	oncology	353	53.40	DN4		No	36 (10.2)	low
Akkar 2016 [24]	Oujda	rheumatology	84	50.6 ± 12.29	DN4		No	26 (31.0)	low
Chahbi 2018 [12]	Marrakech	diabetology	300	57.24 ± 9.79	DN4	4	No	46 (15.4)	low
Tibar 2018 [20]	Rabat	neurology	117	60.77 ± 11.36	DN4	3	No	11 (13.0)	moderate
Ben Lafqih 2022 [13]	Marrakech	obesity	111	44.79 ± 14.75	DN4		No	65 (59.0)	low
Mboussi 2022 [14]	Marrakech	rheumatology	21	52.14	other			2 (9.5)	low
Mougui 2023 [15]	Marrakech	rheumatology	195	59.15	DN4	4	Yes	109 (55.9)	moderate
Guedari 2023 [25]	Casablanca	diabetology	623	51.40	DN4	4	No	138 (22.2)	high
Ben Lafqih 2023 [16]	Marrakech	diabetology	231	45.77	DN4	4	No	65 (28.1)	low
Chakdoufi 2023 [26]	Rabat	diabetology	240	54.00	DN4		No	92 (38.2)	moderate
Fouzia 2023 [23]	Tanger	rheumatology	178	58.0 ± 9.19	DN4	4	Yes	57 (32.7)	moderate
Echchad 2023 [27]	Rabat	diabetology	250	55.20 ± 14.80	other			71 (28.4)	low
Idrissi Ouali 2023 [21]	Fès	rheumatology	3080		DN4	4	No	105 (3.4)	moderate
Douali 2023 [17]	Marrakech	diabetology	85	58.32 ± 10.89	DN4	4	No	34 (40.0)	moderate
El Azizi 2023 [22]	Fès	diabetology	248	52.27 ± 12.00	DN4	4	No	53 (21.4)	moderate
Tebaa 2023 [18]	Marrakech	rheumatology	181	51.00 ± 13.00	DN4			33 (31.0)	moderate

## Supplementary Materials

The following supporting information can be downloaded at: https://www.mdpi.com/article/10.3390/life15050780/s1, Queries for PubMed, Scopus, and Web of Science (WoS) focusing on retrieving studies about the prevalence of neuropathic pain and related terms with Moroccan affiliations.
