# Supplementary material for: Prevalence of Neuropathic Pain in Morocco: A Systematic Review and Meta-Analysis
Source: Life (Basel). 2025 May 14;15(5):780. doi: 10.3390/life15050780 (PMC12112904; doi:10.3390/life15050780)
Supplement: Supplementary file 1 [file life-15-00780-s001.zip › life-3520743-supplementary.pdf]

SUPPLEMENT FILE

PubMed Query:

```
((Morocco[Affiliation]) AND (prevalence[Title/Abstract]))
AND ((((nerve pain[Title/Abstract]) OR (neurodynia[Title/Abstract]))
OR (neuropathic pain[Title/Abstract])) OR (Neuralgia[Title/Abstract]))
```

Scopus Query:

```
TITLE-ABS-KEY(prevalence)
AND TITLE-ABS-KEY("nerve pain" OR neurodynia OR "neuropathic pain" OR
neuralgia)
AND AFFILCOUNTRY(Morocco)
```

Web of Science (WoS) Query:

```
TS=(prevalence)
AND TS=("nerve pain" OR neurodynia OR "neuropathic pain" OR neuralgia)
AND CU=Morocco
```
